# Large-scale image region documentation for fully automated image biomarker algorithm development and evaluation

**DOI:** 10.1117/1.JMI.4.2.024505

**Published:** 2017-06-07

**Authors:** Anthony P. Reeves, Yiting Xie, Shuang Liu

**Affiliations:** Cornell University, School of Electrical and Computer Engineering, Ithaca, New York, United States

**Keywords:** image region documentation, large-scale image documentation, automated image analysis, image analysis system, low-dose chest CT

## Abstract

With the advent of fully automated image analysis and modern machine learning methods, there is a need for very large image datasets having documented segmentations for both computer algorithm training and evaluation. This paper presents a method and implementation for facilitating such datasets that addresses the critical issue of size scaling for algorithm validation and evaluation; current evaluation methods that are usually used in academic studies do not scale to large datasets. This method includes protocols for the documentation of many regions in very large image datasets; the documentation may be incrementally updated by new image data and by improved algorithm outcomes. This method has been used for 5 years in the context of chest health biomarkers from low-dose chest CT images that are now being used with increasing frequency in lung cancer screening practice. The lung scans are segmented into over 100 different anatomical regions, and the method has been applied to a dataset of over 20,000 chest CT images. Using this framework, the computer algorithms have been developed to achieve over 90% acceptable image segmentation on the complete dataset.

## 1 Introduction

Fully automated evaluation of image biomarkers offers the possibility of greatly enhancing the impact of medical imaging by providing detailed information on a patient’s health status. Medical images, especially those that are obtained periodically in the context of screening, provide a rich source of information for disease detection and health monitoring. The opportunity exists for automated image analysis to provide a standard set of quantitative image biomarker values that may be directly used for patient health assessment. This is similar in concept to a single blood extraction that can directly provide a set of quantitative biomarker values through standardized laboratory assays; however, in this case, only the application of the analysis software to existing image data is required. As with the blood test, any findings by an automated image analysis system require follow-up involving a rigorous review by a physician of all available data. Therefore, to be clinically useful it is important that such systems minimize the number of false positives while still maintaining high sensitivity.

The large dataset size required for the new deep learning methods is illustrated in the context of diabetic retinopathy diagnosed from eye fundus images in a recent study by Gulshan et al.^1^ In this study, the training set consisted of 128,175 cases [two-dimensional (2-D) images]; additionally, two independent test sets that involved a total of 11,711 cases were used. In contrast, a survey of eye-fundus image systems using traditional image analysis methods^2^ reported on 26 different studies on diabetic retinopathy characterization. The number of cases used in these studies had a range from 20 to 16,770 with a median of 250.

To achieve the high algorithm robustness necessary for unsupervised computer operation and to meet the modern machine learning method training requirements, very large documented image datasets that contain many more cases than what is typical for current academic studies are needed. In this paper, a method and process for creating a large documented dataset for the purpose of robust segmentation of quantitative image biomarkers are described. This method has been used over four annual cycles to (a) increase the number of documented cases in the dataset from 364 to 20,749 and (b) improve the robustness of image analysis algorithms on this large dataset to a greater than 90% overall success rate on automated quantitative image biomarker evaluations.

The contributions of this paper are:

1. A method for visually evaluating the segmentation of image region based on target quantitative image biomarker tasks that are scalable to very large image datasets.
2. A process that minimizes the frequency of visual inspections to improve scalability and does not require any manual image annotations.
3. The presentation of a multiorgan segmentation scheme for the analysis of quantitative image biomarkers in low-dose chest CT (LDCT) images.

The outcomes of using this image documentation process in our academic research lab environment to develop robust automated image segmentation algorithms are reported.

### 1.1 Chest Low-Dose CT Image Analysis Demonstration Application

The methods for dataset documentation are presented in the context of the analysis of LDCT three-dimensional (3-D) images that are acquired for lung cancer screening (LCS). With the recent approval of LCS,^3^ a large at-risk population of over 8 million patients in the United States is eligible to receive annual LDCT 3-D images. This population is also at high risk for many other diseases in the chest, and the LCS provides an opportunity for annual monitoring. The chest health analysis system (CHAS) system currently evaluates image biomarkers for the lungs, heart, breast, and major vessels and major bone structures. Lung cancer screening CT 3-D images are designed for the detection of pulmonary nodules and have the following characteristics: coverage of the whole lung region, acquired in a single breath-hold, low-dose, and thin slice (1.25 mm or less for current protocols). In LCS, the primary objective is to identify pulmonary nodules. The high image contrast between these nodules and the surrounding lung parenchyma enables radiologists to use a low-dose CT protocol that minimizes radiation exposure to the patient. However, the consequence of using this low-dose protocol is that the image noise, which is inversely related to radiation exposure, is much higher than in typical clinical CT scans; this is especially relevant to nonlung regions of the image.

A database of over 20,000 documented LDCT 3-D images has been created over the last 5 years to aid the development of a fully automated CHAS.^4^^,^^5^ This system automatically evaluates a set of quantitative image biomarkers and involves multiple region segmentations of LDCT 3-D images. The CHAS was originally designed to identify pulmonary nodule candidates to assist the radiologist in the lung cancer-screening task; it has been extended to report on other diseases and organs that are imaged by these scans. The current CHAS automatically identifies over 100-labeled segmented regions in LDCT 3-D images with a slice thickness of 2.0 mm or less.

### 1.2 Fully Automated 3-D CT Image Segmentation Evaluation

Fully automated algorithms must be validated on a very large number of test cases before they can be approved for general clinical use. Evaluation cases must well represent the spectrum of clinical presentations. These requirements are in contrast to most traditional image biomarker studies reported in the research literature. Those studies typically involve very small selected datasets, and, further, they employ semiautomated computer methods that require physician interaction. We review here the evaluation methods and number of images employed for the main studies that are most closely related to our CHAS demonstration application.

A number of fully automated image segmentation systems have been reported in the literature. Segmentation performance is usually reported by subjective *post hoc* visual evaluation (VE) or by quantitative comparison to a small number of pre-established expert manual markings (QE). For QE, two popular variations that reduce the manual effort required are automated QE (QEa) in which a semiautomated method with manual corrections is used for image markings and sampled QE (QEs) in which only a subset of 2-D slices in a 3-D image are marked and evaluated.

Most work in this area has been performed for a single organ class and has been validated with a limited number of test cases. Very few studies of LDCT 3-D images that meet the recent requirements for LCS have been done.^6^ Studies that involve chest and body CT segmentation are listed in Table 1. To be state-of-the-art, we have included studies that do not conform to the LCS requirements; and to represent multisegmentation studies, we have included CT studies for the abdomen. Studies in other 3-D image modalities typically have similar characteristics. Our main interest is in thin-slice, low-dose thoracic scans that are relevant to LCS; most of the listed studies are thin-slice but not low-dose. In the column for the number of cases, we list any variations from our desired protocol. Since very few studies use LDCT, they are specifically identified; otherwise the study includes regular dose CT 3-D images. The size in mm when mentioned is the slice thickness of the scans. Some studies provide QE for a small number of cases only due to the effort required and VE for a larger number of cases. Only studies with more than 25 CT scans in the evaluation set have been included in Table 1.

**Table 1:** Anatomical segmentation studies of body regions in CT 3-D images.

First author	Year	Organ	Number of cases	Evaluation
Leader^7^	2003	Lungs	101 (10 mm)	QEa
Zheng^8^	2003	Lungs	55 (7 to 10 mm)	QEs
Farag^9^	2010	Lungs	50 (LDCT)	QE
Gill^10^	2014	Lungs	212	QEa
Xu^11^	2014	Lungs	400	VE
Zhang^12^	2006	Lung lobes	29 (7 EBCT: 3 mm)	QEs
Lassen^13^	2013	Lung lobes	75	QEs
Pu^14^	2009	Lung lobes	65	VE
Kurkure^15^	2008	Aorta	37 (EBCT: 3 mm)	QE
Isgum^16^	2009	Aorta, heart	29 (LDCT)	QEs
Xie^17^	2013	Aorta	359/60	VE/QEs
Xie^18^	2014	Heart	400	VE
Xie^19^	2015	Pulmonary trunk	347/45	VE/QEs
Zhou^20^	2008	Breast	66 (torso CT)	QEa
Liu^21^	2016	Breast	1270	VE
Liu^22^	2015	Sternum	351/50	VE/QEs
Lee^23^	2010	Ribs	115 (LDCT)	VE
Yao^24^	2006	Vertebra	71(5 mm)	VE
Naegel^25^	2007	Vertebra	26 (abdominal CT)	VE
Kim^26^	2009	Vertebra	50 (abdominal CT)	VE
Klinder^27^	2009	Vertebra	64 (CT)	QEa
Haas^28^	2008	Multiorgan	302 (chest CT) + 260 (pelvic CT)	VE
Okada^29^	2012	Multiorgan	28 (abdominal CT)	QE
Chu^30^	2013	Multiorgan	100 (abdominal CT)	QEa
Oda^31^	2012	Multiorgan	100 (abdominal CT)	QE
Zhou^32^	2014	Multiorgan	1000 (torso CT)	VE
SLIVER challenge^33^	2007	Liver	40 (abdominal CT)	QEa
EXACT09 challenge^34^	2009	Airway	40 (LDCT)	QEa
			10 fully automated methods	
LOLA11 challenge^35^	2011	Lung	55	QE
			14 fully automated methods	
VISCERAL challenge^36^	2012 to 2016	Multiorgan	30 (whole body CT) +30 (trunk CT)	QE
			20 fully automated methods	

Most segmentation studies reported in this chest CT literature are based on less than 100 cases. Other than our own work, VE studies involve a median of 71 cases (min=26, max=1000) and QE studies involve a median of 55 cases (min=28, max=212). Beyond our own work, only three studies address the much more challenging problem of segmenting low-dose 3-D images^9^^,^^16^^,^^34^ and only four studies reported significantly more than 100 cases (Gill et al.^10^ 212, Xu et al.^11^ 400, Haas et al.^28^ 302, and Zhou et al.^32^ 1000). For fully automated algorithm design and evaluation, much larger datasets are needed.

One approach to comparing algorithms is through the process of challenges^33^ where several algorithms for a task are evaluated on a common dataset. Four of the studies listed in Table 1 are challenges.^33^^–^^36^ The visceral challenge group has, to date, conducted various organ segmentation challenges using the QE method. They have recently proposed a method for large image documentation^36^^,^^37^ based on atlas registration to expert annotated reference cases and label fusion from multiple algorithms to improve segmentation quality. A recent paper by Dicente Cid et al.^38^ involves lung segmentation on a dataset of 12,414 CT scans using the difference between two lung segmentation algorithms to identify problem segmentations. By requiring a 0.95 Dice coefficient agreement between the two algorithms, they rejected 35% of the 3-D images. While this approach allowed preliminary results to be obtained on a large dataset, a large fraction of the data was rejected and it is unknown how many of the accepted segmentations had serious issues since no visual review was performed.

Both the VE and QE approaches suffer from significant drawbacks; the main drawback is the amount of time required to review each image. VE may be applied to a large number of cases, but it is highly subjective and is very costly to repeat (same cost as first evaluation), which must be done for each cycle of evaluation (i.e., for each algorithm modification); further, repeatability is an issue due to the subjective evaluation. QE has the advantage of repeatability. Since a target “ideal” segmentation is created, the evaluation is quantitative and may be automated. However, two issues with this approach are: (a) the time per image to create the target segmentation manually is very large and the “ideal” segmentation has built-in human variation. Frequently QEa and QEs variants are used to reduce this time; however, these techniques introduce new errors due to algorithm bias (QEa) or reduced precision by subsampling the data (QEs).

The NCI sponsored Lung Image Database Consortium^39^ (LIDC) created a public image database of 2669 pulmonary nodules in 1018 chest CT 3-D images. QEa evaluation was used with four experienced radiologists marking the boundary of each nodule. The very high intermarker variation^40^ illustrates the issues with the QEa method and makes the use of these markings for precision evaluation of computer algorithms challenging. A significant fraction of the multimillion dollar project cost was for the 3-D image marking process, which only addressed documenting a tiny fraction of the pixels in these 3-D images.

### 1.3 Image Segmentation Errors

Segmentation errors may be considered to fall into two general classes: precision errors ($E_{P}$) and catastrophic errors ($E_{C}$). Precision errors occur due to differences in details among algorithms or between algorithms and the variation of manual image annotations: for this error type the difference among methods for large organs is typically small. Catastrophic errors occur when an algorithm incorrectly identifies or includes a significantly different region with the target region (for example, includes a nearby vessel as part of a lesion); the size of these errors may be very large. A catastrophic error $E_{c}$ is defined for a 3-D segmentation as a connected incorrect region of a segmentation that involves less than 50% of the surface area of the true image region (typically much less than 50%) and causes a significant change in the overall segmentation volume or spatial extent compared to the true image region. In most studies on segmentation algorithms, the dataset is usually small (<100 cases) and of carefully selected images such that the majority of the errors tend to be $E_{P}$. However, when larger datasets with a wider range of imaging parameters and presentations are considered, the likelihood of $E_{C}$ errors is significantly increased. In a semiautomated environment where the primary target is image region characterization, $E_{C}$ errors are rarely an issue since the operator manually corrects them. However, for a fully automated system, the objective has a focus on abnormality detection rather than characterization and $E_{C}$ errors are a major consideration since they may cause an unacceptable large number of false-positive abnormality detections. Similarly, $E_{C}$ errors may also adversely affect sensitivity if the whole region is not included in the segmentation. The evaluation criterion we use for image segmentations in this case primarily relates to the $E_{C}$ error type.

In the large-scale image documentation method, we introduce the concept of 3-D image visualizations for segmentation evaluation, which provides a rapid image review that is primarily sensitive to the $E_{C}$ error. We also use a simple two- or three-level categorical evaluation that is targeted to biomarker evaluation requirements rather than minimizing $E_{P}$ errors, which is expected to have high interreader agreement.

Recent studies in large-scale image datasets have had to deal with the significant number of problem cases in these datasets. In the study by Gulshan et al.,^1^ 10.1% of the images were excluded for evaluation by poor quality evaluation; in the study by Dicente Cid et al.,^38^ 35% of the images were excluded by algorithm comparison. In contrast, in our study, less than 0.5% of the cases are rejected for quality issues. Consequently, we have a significant number (our target is <10%) of images for which we do not have acceptable segmentations. One approach would be to use manual methods to correct the algorithm outcomes; such an approach has been implemented by Takx et al.^41^ in which they established a database with 1749 validated biomarker outcomes. Experienced physicians manually correct outcome regions for calcium scoring. Although our system has the capability for manual correction, we did not adopt this approach due to the very large burden it would place on human resources for the large image regions we are concerned with and because such a process would introduce human variation into the image documentation.

The image documentation system, presented here, addresses the main shortcomings outlined above. A simple categorical grade related to biomarker evaluation is employed to address the segmentation precision issue. Human variation is addressed by not using any manual markings. Finally, VE evaluation time is minimized (a) through the use of customized visualizations and (b) by minimizing the number of images to review through the use of automated quantitative preselection criteria. This documentation method has been used to create a documented image database with over 20,000 cases and facilitates fully automated algorithm evaluation on multiple segmentations.

### 1.4 Image Documentation Implementation

The methods for large dataset documentation presented here have been implemented and evaluated on the system for image management and biomarker analysis (SIMBA).^5^ While image segmentation studies have been ongoing since 2000, the first instance of the formal framework presented here was implemented in 2012. The data documentation system is updated each year by installing the latest version of our image analysis software as the reference documentation method and updating the documentation for all images in the database with this method. There have been five annual revisions of the system, with the outcomes of last two (2016 and 2017) reported here. The SIMBA system^5^ is a mature web-based image management platform for research studies including: study data collection and analysis,^42^ image-based clinical studies,^43^ public image databases,^44^ and image analysis algorithm development.^17^^,^^45^^,^^46^ The concept of SIMBA is to have all image data and study data in a single web-based management system. It was first used in the I-ELCAP study for data collection and analysis starting in 2000. The system has the capability for full medical image review and annotation and includes the relevant functions of a PACS system through a web interface. For the LIDC study;^39^ it was the only one of the image annotation tools used by the five participating institutions that was web-based.

### 1.5 Overview

The methods section contains three components: the details of the large-scale image documentation system, the CHAS demonstration application, and the documentation evaluations. Key aspects of the method are: a new process for image evaluation, VE with quantitative review (VEQR), and the employment of customized segmentation visualizations. The documentation of over 20,000 3-D images with the SIMBA image documentation system is discussed.

## 2 Methods

The system for image documentation realizes three basic operations: algorithm evaluation, documentation database revision as a result of the outcomes of evaluating a new algorithm, and the addition of new image data. Algorithm evaluation involves visual review of the differences between an algorithm’s outcome and the database documentation. Database revision involves updating the database documentation when a new algorithm provides superior outcomes to the current documentation. New image data are documented by visual grading of the outcomes of reference segmentation algorithms. Some algorithm outcomes receive an unacceptable segmentation grade, which means that the associated segmentation is not of sufficient quality to be used for biomarker evaluation; the images corresponding to these outcomes may be used to construct worst-case test sets to assist in algorithm development.

Traditional segmentation algorithm development begins with a classical study involving the order of 100 cases with expert marking or review. Then, algorithms are further developed on a development set of 364 cases (available from public image databases).

The goal of the large-scale image documentation is to facilitate the development of highly robust computer algorithms that rarely fail to provide useful outcomes, including algorithms based on modern machine learning methods. The image documentation system organization is discussed in Sec. 2.1; then the method for VE is presented in Sec. 2.2.

### 2.1 Image Documentation System Organization

**Fig. 1:**
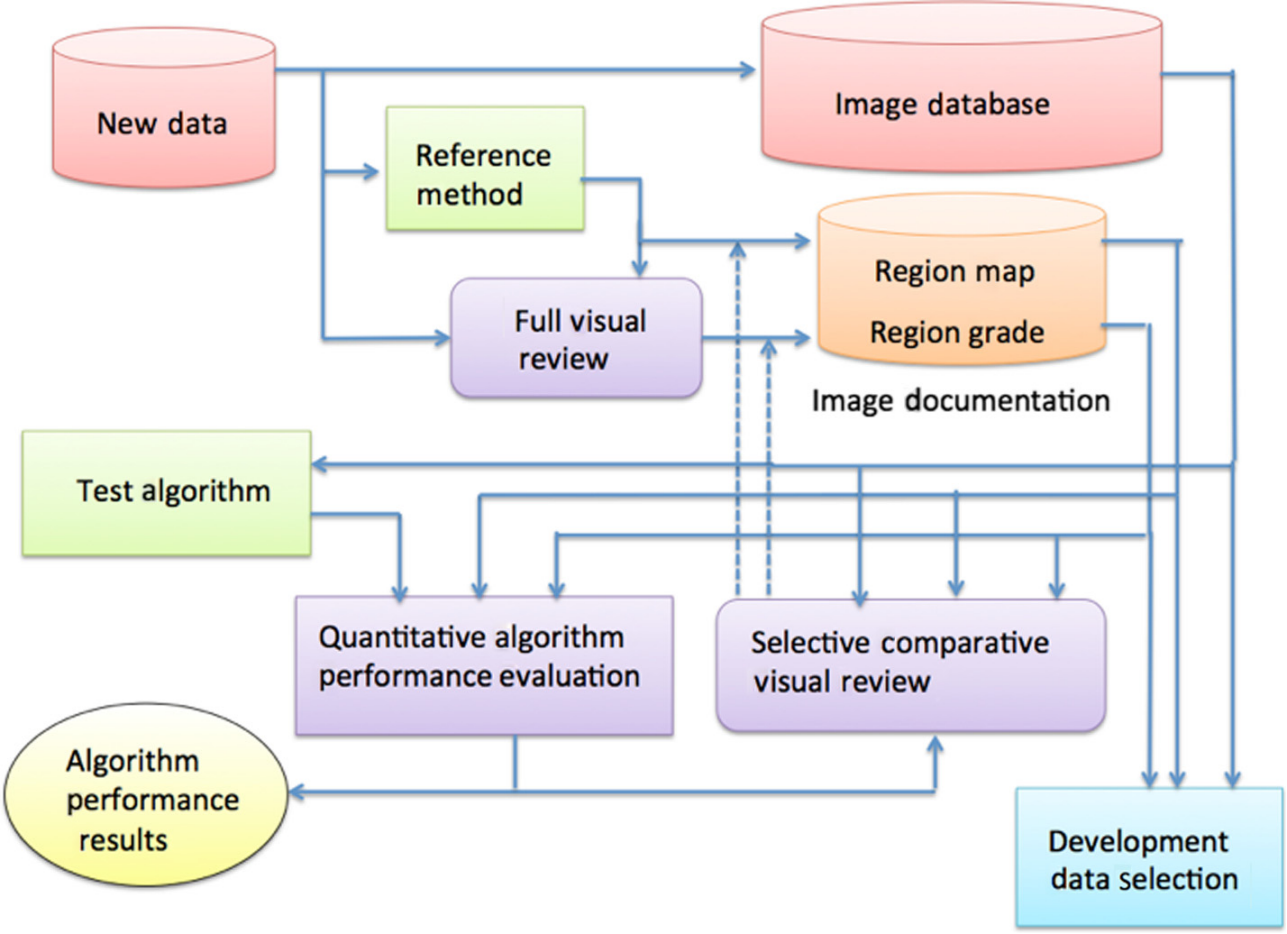
The SIMBA framework for segmentation algorithm development and evaluation on large medical image datasets.

An overview of the SIMBA system for large-scale image documentation is illustrated in Fig. 1. The database within the system comprises two main components: the image database in which the raw DICOM format images are stored and the data documentation. The data documentation has two components for each image entity: a label map, which contains a label (a unique segment identifier) for every pixel in the corresponding raw image, and a label score set, which contains a categorical grade for every label that has been identified for that image.

The four main framework operations are (a) addition and evaluation of new image data, (b) automated algorithm evaluation, (c) data documentation updating, and (d) the selection of datasets for algorithm development. The key components of the framework include:

1. Documented image database: This database comprises three main storage components: raw DICOM images, region label maps for each image, and a set of grades for each label region.
2. Reference method: This is a well-tested algorithm or set of algorithms that does a fully automated segmentation of the image data and produces a label map containing a unique label value for each pixel. This algorithm is used to establish the initial baseline documentation for new images.
3. Full visual review: One or more experienced reviewers observe the customized visualizations for the different segmentations and, possibly, the original image data with the segmentation. Each main segmentation is given a visual grade, which is stored with the corresponding label map in the image documentation.
4. Quantitative performance evaluation: The segmentation produced by an algorithm being evaluated is compared to the corresponding segmentation in the image documentation and the outcome for a set of test images is recorded.
5. Selective comparative visual review is used to update the image documentation. New segmentations produced by the test algorithm that meet the matching criteria are presented to the expert reviewer as side-by-side customized visualizations. The reviewer determines if an update to the region and the grade in the image documentation is to be made.
6. The development data selection accesses the image documentation and selects image cohort lists that are used for the development, evaluation, and validation of new segmentation algorithms. Any partitioning of images, for example for testing and training sets etc., are managed by this component.

The detailed operations are as follows:

a. Addition of new image data: New image data are added directly to the image database; in addition, the image is segmented by the reference method. The outcome is visually reviewed and graded. The segmentations and their grades are recorded in the image documentation.
b. Automated algorithm performance evaluation: A segmentation algorithm (or set of algorithms) to be evaluated is applied to a list of images, and the outcomes are compared to the image documentation. The results for all test images are collected for statistical analysis. Information to be reported depends on the application; options include: overall performance scores, e.g., numbers of good and acceptable segmentations; DC values for each case; and, for development, the images and corresponding segmentations for selected cases. If the image documentation is updated from the outcomes of the test algorithm by comparative visual review, then the performance evaluation should be considered a second time with the updated image documentation.
c. Image documentation updating: When a new algorithm is evaluated, if the outcomes are significantly different from the current image documentation, then these differences may be evaluated by visual inspection and the image documentation may be updated (see the dashed arrows in Fig. 1). The automated performance evaluation and image documentation updating are also used to facilitate periodic updates to the reference method.
d. Identification of development datasets: By searching the grades in the image documentation, it is possible to identify subsets of images for which either segmentation is not yet known or for which the grade is not high. These cases make ideal worst-case examples for the development of improved algorithms.

#### 2.1.1 Segmentation visual quality grading

In the fully automated context, each segmentation is visually categorized into one of two primary categories of “good” and “unacceptable;” the unacceptable category is caused by an $E_{C}$ error. In some cases, we have also defined a third category of “acceptable.” The quality grading is based on the suitability of the segmentation to be used for the evaluation of quantitative image biomarkers. The three grade categories are defined as follows:

1. Good: The segmentation has no major visible errors.
2. Acceptable: The segmentation has visible defects, but these are not expected to impact the resulting quantitative image biomarker values.
3. Unacceptable: The segmentation is inadequate for a reliable evaluation of the corresponding quantitative image biomarker.

Segmentation “truth” is signified by regions with good and acceptable grades. The unacceptable grade identifies cases for which algorithm development is required. Not all segmentation algorithms have an “acceptable” grade. For example, for lung segmentation, the segmentation quality must be good to evaluate whole lung biomarkers such as lung volume and emphysema index. In contrast, in coronary calcium scouring, the heart region may “leak” a little into the surrounding region if the correct calcifications within that region can still be reliably identified.

In our documentation method, there are two instances where visual inspection occurs. In the first full image review, the reviewer simply assigns a segmentation grade. In the side-by-side, selective comparative visual review comparing a new segmentation to the current database version, the reviewer grades the new segmentation and selects the better of the two segmentations even if the grade is not changed from acceptable to good.

The details of this approach are described in the next section.

#### 2.1.2 Validation by visual evaluation and quantitative revision

The VEQR^1^ method is employed to address the evaluation of segmentation algorithms. The VEQR method is described with respect to the VEQR database D and its associated operations. In general, customized visualizations are also used to optimize the visual review efficiency.

The validation database D consists of the following four components:

1. Image set I
  I = {i|i is an image to be segmented and analyzed}
2. Label map set L
  L={l(i)| ∀ i∈I},
  where l(i) is a label map of the same dimensions as image i and the value of each l(i) voxel represents the label value that indicates to which segmented region the corresponding voxel belongs. For instance, a label value of LungR indicates that the respective voxels in image i belong to the right lung region, and a label value of LungL indicates that the respective voxels belong to the left lung region, where both of the labels are assigned by the lung segmentation algorithm.
3. Label assessment set A
  A={a(i,s)| ∀ i∈I, ∀ s∈S},
  where a(i,s) is the quality grade determined by the visual assessment for a specific segmented region s in image i; S is the set of regions that can be segmented; for instance in the CHAS application, S = {lung, airway, rib, vertebra, skin, cardiac region, and breast}. The quality grade must have at least two values: acceptable and unacceptable. For the CHAS application, three values are used a(i,s)∈ {good, acceptable, and unacceptable}.
  Note that the set of segmented regions, S, is not equivalent to the set of segmentation labels in the label map. In general, several subregions with distinct label values constitute a single segmented region. For example, the segmented region rib corresponds to 24 subregion labels (ribL1,ribL2,…,ribL12, ribR1,ribR2,…,ribR12) in the label map; the segmented cardiac region corresponds to three labels (heart, aorta, or pulmonary trunk) in the label map. The grade a(i,s) is determined according to the overall quality of the segmented region s. If the region contains subregions, then each subregion is graded individually and the overall grade is set to the lowest subregion grade.
4. Reference algorithm set R
  R={r(s)| ∀ s∈S},
  where r(s) is an algorithm that segments target region s and it is applied to any new images added to the database to establish the initial label map and label assessment; S is the set of segmented regions as described above.

The validation database supports three main functions: algorithm evaluation, new image addition, and database revision.

1. Algorithm evaluation is a fully automated operation that provides quantitative performance measurement of a new algorithm by comparing its outcome to the reference segmentation saved in the label map. Consider a target region s and a new algorithm n(s) for the segmentation of s; the aggregate performance score for n(s) is determined on the evaluation on a subset $I_{\mathrm{EV}}$ of the image set I, where $I_{\mathrm{EV}}$ = {i| ∀ i∈I and a(i,s) = good or acceptable}, i.e., only images with good or acceptable reference segmentation of s are used to evaluate the new algorithm. Given an image $i\in I_{\mathrm{EV}}$, if we let $l_{S}(i)$ denote the reference segmentation of s, which can be extracted from label image l(i), and let $l_{s}^{n}(i)$ denote the segmentation generated by the new algorithm n(s), then the Dice coefficient (DC) can be computed as follows to serve as the quantitative comparison measurement of the two segmented regions:
  $$\mathrm{DC}[l_{S}(i),l_{s}^{n}(i)]=\frac{2|l_{s}(i)\cap l_{s}^{n}(i)|}{\left|l_{s}(i)|+|l_{s}^{n}(i)\right|}.$$
  The DC values are averaged among all images in $I_{\mathrm{EV}}$ to serve as an aggregate performance score for the new algorithm n(s). In future evaluations, we plan to use a locally sensitive measure such as the Hausdorff distance in addition to the DC measure to provide additional sensitivity to small local errors.
2. Database revision is an update to the database documentation based on the outcomes of a new algorithm n(s) that provides superior segmentation outcomes for some images compared to the current reference segmentation. Given an image i∈I and a target segmentation region s, the segmentation outcome $l_{s}^{n}(i)$ generated by the new algorithm is first compared with the reference segmentation $l_{S}(i)$ by computing the $\mathrm{DC}[l_{S}(i),l_{s}^{n}(i)]$ as defined above. Then, the database revision is conducted as follows:
  a. If $i\in I_{\mathrm{EV}}$ and the DC is less than a preset level $T_{\mathrm{DC}\text{low}}$, then the new segmentation for that image is considered to be inferior to the reference segmentation; thereby, no update is made.
  b. If $i\in I_{\mathrm{EV}}$ and the DC is greater than a preset level $T_{\mathrm{DC}\text{high}}$, then the new segmentation is not considered as a significant improvement over the reference segmentation; thereby, no update is made.
  c. For the remainder of the cases, visual inspection is required. If the new segmentation $l_{s}^{n}(i)$ is considered to be superior to the reference segmentation $l_{S}(i)$, then the label map l(i) is updated by replacing the respective segmented region with the outcomes of the new algorithm. Any improvement in grade is also recorded.
3. New image addition: When a new image $i_{n}$ is added to the database, each algorithm from the reference algorithm set R is applied to $i_{n}$ and the outcomes are visually evaluated. The database D is then updated correspondingly by adding the new image $i_{n}$ to the image set I, the label image $l(i_{n})$ to the label image set L, and the quality grade $a(i_{n},s)$ for each segmented region s to the label assessment set A, respectively.

For the most precise results for a new algorithm evaluation, the database should first be revised by that algorithm before it is evaluated; however, that involves a cycle of visual inspection, and other algorithms would need to be evaluated on the new database for performance comparisons. The algorithm performance is then rated as the fraction of positive evaluations in the database to the total number of images in the database, including those without successful region documentations. For algorithm development, it may be useful to select a subset of the database for evaluation; typically this subset is made of cases for which the segmentation is rated as acceptable or unacceptable. It is possible to sequester a partition of the database for blind algorithm evaluations if necessary.

#### 2.1.3 Customized visualization for segmentation grading

**Fig. 2:**
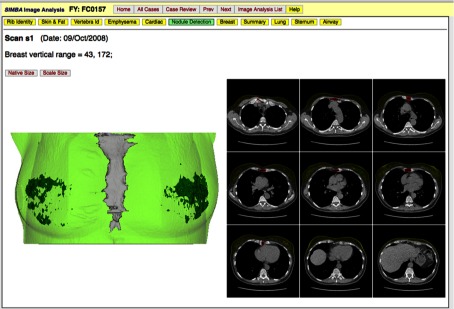
Snapshot from SIMBA of a 3-D visualization of a breast region segmentation.

Customized 3-D visualizations are used to minimize the time required for VE. Algorithm outcomes are graded for a score a(i,s) by a two-stage VE process. A 3-D image is first evaluated by a 3-D customized visualization, and, for additional review when necessary, a more traditional 2-D image slice viewing is provided. Efficient VE is especially important for 3-D image modalities since the traditional approach of examining a region on each 2-D slice through the 3-D image is very labor intensive. The 3-D visualization is developed in concert with the early development of the corresponding segmentation algorithm. The visualization web page is developed with experience gained from observation of the most common segmentation errors. A snapshot for the customized 3-D visualization of the breast region in chest CT 3-D images is shown in Fig. 2.

In this study, the custom visualizations were developed by the computer algorithm developers following the initial validation of the algorithm by clinical experts. Thus, the 3-D visualizations and accompanying selected 2-D image views were designed to be sensitive to known-algorithm errors. The 3-D visualizations were developed using the custom visualization tools in the V4 VisionX software system^47^ that uses the Visualization Toolkit^48^ for image rendering.

**Fig. 3:**
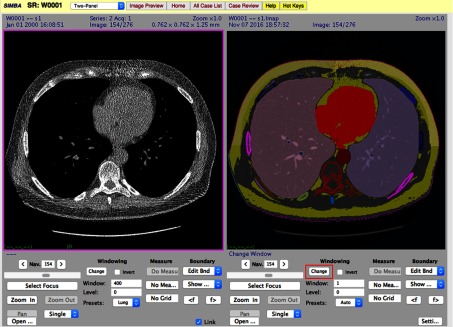
SIMBA snapshot of a traditional 2-D image slice review with segmentation map.

In addition to the 3-D visualization of the breast and the fibroglandular tissue, there are 9 additional 2-D image slices with regions marked as shown on the right side of Fig. 2; these have been found to be useful for resolving many potential issues observed on 3-D visualization. If the reviewer is not satisfied with the first level 3-D review, then a second traditional review is available as shown in the snapshot in Fig. 3. This review permits standard windowing and zooming etc. for a side-by-side comparison of the original 2-D image slices and 2-D image segmentations.

**Fig. 4:**
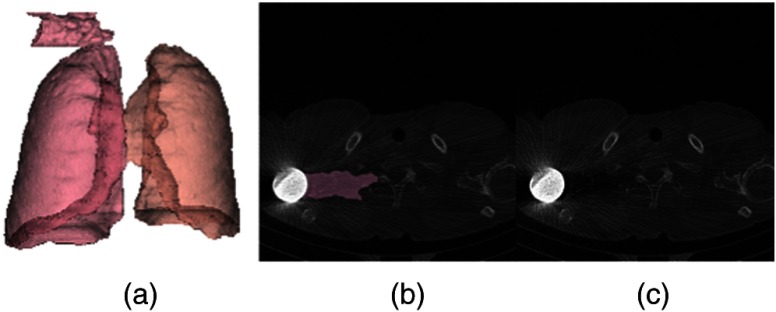
Example unacceptable lung segmentation (a) segmentation shown in 3-D, (b) segmentation shown in 2-D, and (c) intensity CT image of the same axial slice.

In the current VEQR method, a region segmented by an algorithm is evaluated by VE into three categories: good (G), acceptable (A), and unacceptable (U). The criterion for acceptable is that, although there is some visible defect in the segmentation, the quality is sufficient for evaluation of related biomarker measurements. An example of an unacceptable segmentation for the lungs is shown in Fig. 4. This is clearly visible in the 3-D visualization alone. (The problem for this case is also visible in the 2-D review, but this is not needed for database documentation).

It is interesting to note that our segmentation and an external segmentation algorithm^49^ had very similar segmentations for this case (DSC=0.98), which illustrates an issue of relying on a method such as label fusion to improve confidence in a segmentation without visual review.

**Fig. 5:**
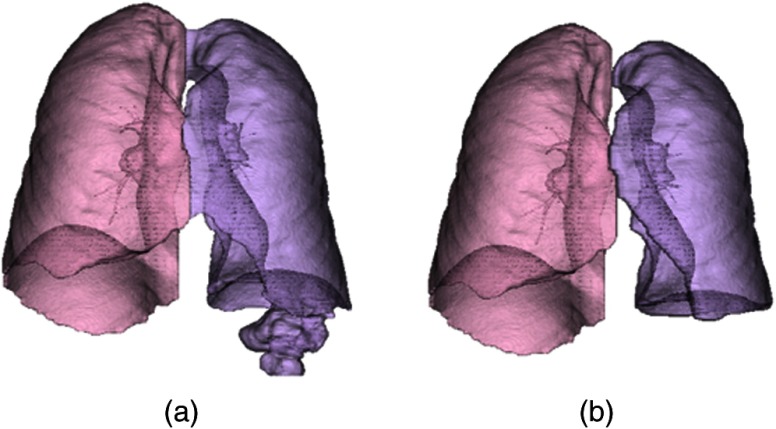
(a) Unacceptable segmentation and (b) acceptable segmentation.

The criteria for grading are based on the ability to precisely compute the quantitative image biomarkers associated with the segmentation. An example of unacceptable and acceptable grades for the same case with two different algorithms is shown in Fig. 5.

In Fig. 5(a), the left lung has leaked into the bowel. This is sufficient to modify the values for the lung quantitative biomarkers for the left lung: lung volume [2124 mL for (a) and 1972 mL for (b)] and emphysema index [2.33% for (a) and 1.41% for (b)].

For a new 3-D image region or quantitative image biomarker, the development of the 3-D visualization follows the traditional validation of the new algorithm. Then, the 364 images of our development set with this visualization are very carefully reviewed by two experienced graders. The image biomarkers computed from these segmentations are also reviewed.

### 2.2 Chest Health Analysis System

**Fig. 6:**
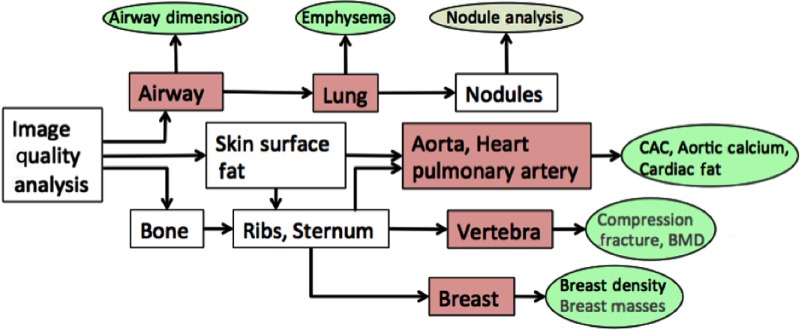
CHAS modules and their dependencies organization. The associated biomarkers are also listed.

The CHAS consists of a number of interdependent anatomy function modules as outlined in Table 2. The models and their dependencies are also shown in Fig. 6. The main anatomy segmentation labels that are managed by each module are also listed in Table 2. The CHAS system employs a divide and conquer approach to the image labeling process in which the most robust labeling algorithms are applied first and subsequent algorithms depend on the existing framework and labeled regions established by earlier algorithms. This approach is appropriate for the LDCT scans in which the high degree of noise makes soft tissue segmentation very challenging.

**Table 2:** Chest analysis functions: module names, descriptions, and their associated main labels.

Module	Description	Main labels
Image quality	Measure calibration and noise in the air outside body and compute profile. Set no data region (outside scan reconstruction circle).	No-data
Airway	Identify lumen of trachea and main bronchi using cylinder tracking.^50^^–^^52^	Trachea, main bronchi
Lung	Left and right lung segmentations: lung regions identified from airway then separated.^46^	Left lung, right lung
Nodules	Nodule analysis, nodule candidates queued for radiologist review.^53^^–^^55^	Nodule candidates
Skin surface	Skin surface, body scan boundary determination	Skin, scan body boundary
Fat	Noise-sensitive fat segmentation.^56^	Fat
Aorta	Aorta segmentation using cylinder tracking and surface refinement.^17^	Aorta: aortic calcium
Heart	The general heart soft tissue region constrained by adjacent anatomical structures.^18^	Heart region: cardiac fat and CAC
Pulmonary artery	Pulmonary artery region between bifurcation and heart surface.^19^	Pulmonary artery
Bone	Bone (calcification) segmentation through intensity thresholding.	Bone
Ribs	Spinal canal tracking.	Spinal canal
	Rib identification and individual labeling by proximity to spinal canal.^23^	Ribs (24 labels)
Vertebra	Spine segmentation, individual vertebra separation, and closed surface segmentation of each vertebral body.^45^^,^^57^	Vertebra (15 labels)
Sternum	Sternum segmentation through tracking in cranial and caudal directions.^22^^,^^57^	Sternum
Breast	Whole breast segmentation and glandular tissues analysis: left and right breast regions are separated based on the sternum location.^21^^,^^58^	Left breast, right breast, gland: tissue

In addition to pulmonary nodule detection (usually the primary reason for obtaining the scan), the system also evaluates a number of other organs that are imaged; these evaluations include, for example, coronary artery calcium (CAC) scoring,^18^ emphysema measurement,^59^ and breast density.^58^

#### 2.2.1 CHAS algorithms

The image analysis begins with the image quality module, which assesses image noise^56^ outside the body as well as various image acquisition parameters (see Fig. 1 and Table 2). This sets the image acquisition and quality profile so that other modules can compensate for specific scan parameters that affect image quality such as the image reconstruction kernel. The region of the image that is outside the reconstruction space is set to the “no-data” label.

For lung health, airways are first labeled^52^ and then the individual lung regions^46^ are identified. From the lung segmentation, the nodule module identifies pulmonary nodule candidates^46^ and the emphysema module computes emphysema indices. The growth rate analysis of pulmonary nodules is currently managed by a semiautomated system with physician interaction and review.

The skin surface segmentation^56^ provides a separation of the body from other objects in the scanner field of view such as the scanner table. For bone analysis, significant calcified structures are first identified, then ribs^23^^,^^57^ and vertebra^45^^,^^57^ are labeled; finally, the sternum^22^^,^^57^ is labeled, as it is used in the breast analysis.^21^^,^^58^ The cardiac analysis^17^^,^^18^ depends on both the bone and lung segmentations. The aorta is segmented^17^ using cylinder tracking, and its profile is measured from the segmented volume. The heart region is localized, and different calcifications (mitral valve, aortic valve, and coronary artery) are identified in this region so that a robust CAC score can be obtained.^18^ In more recent work, the vertebral bodies are segmented^45^ to identify osteoporosis and compression fractures, and the breast region is segmented^21^^,^^58^ to evaluate breast density and identify any breast abnormalities. The current system embodies 110 individual pixel labels plus a set of group labels. This number is rapidly increasing as new algorithms are developed.

#### 2.2.2 CHAS quantitative image biomarkers and the analysis report

Once segmentation has been completed, an analysis report that contains quantitative measurements based on the segmented regions is generated; for example, an emphysema score based on the left and right lung segments is generated. Standard established measures we report include: airway dimension (diameter and wall thickness), emphysema score, cardiac calcium and fat content, and breast density. In addition, we continue to research and report more recently defined biomarkers. The reported image biomarkers are listed in Table 3 together with the segmentation module that must successfully complete for their evaluation.

**Table 3:** Image biomarkers by CHAS.

Image biomarker	Item	Segmentation dependency
Airway lumen diameter^50^	Average diameter (mm) for each detected segment	Airway
Airway wall thickness^50^^,^^51^	Average distance (mm)	Airway
Pulmonary nodule detection^53^^–^^55^	Location (x,y,z) and size (mm)	Lung
Pulmonary nodule measurement^46^	Size (volume ${\mathrm{mm}}^{3}$)	Lung
Pulmonary nodule pair measurement^46^^,^^60^	Growth rate (volumetric growth index)	Lung
Pulmonary nodule characterization^60^^–^^63^	Status (malignancy probability)	Lung
Emphysema measurement^59^	Emphysema index lung volume (ml)	Lung
CAC^18^	Agatston, volume, and mass score	Aorta, heart
Aortic calcium^64^	Agatston, volume, and mass score	Aorta, heart
Pulmonary artery trunk^19^	Pulmonary trunk diameter, pulmonary trunk to ascending aorta diameter ratio	Pulmonary artery
Cardiac visceral fat^65^	Cardiac visceral fat volume and its ratio to heart region volume	Heart, fat
Breast density^58^	Glandular tissue volume to whole breast volume ratio	Breast

The customized visualization and grades criteria are described in detail below for the regions associated with image biomarkers listed in Table 3: airway, lungs, vertebra, cardiac region, and breast region.

#### 2.2.3 Evaluation criteria and customized visualizations

##### Airway

**Fig. 7:**
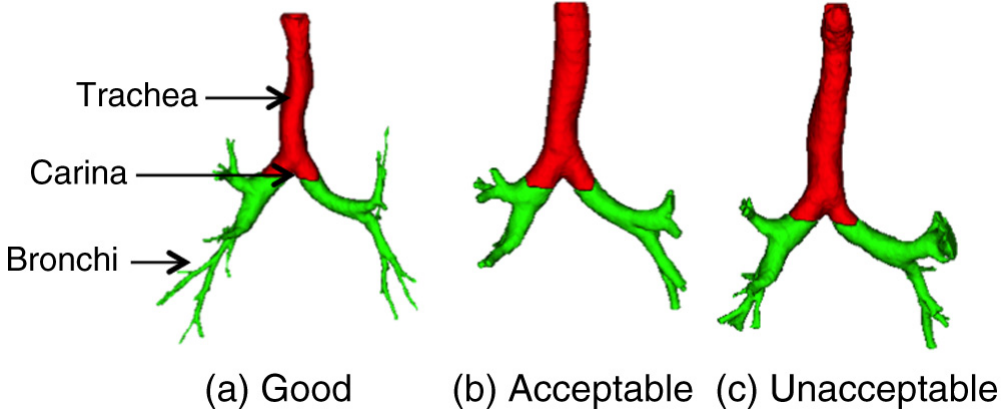
Customized visualization for airway tree segmentation: trachea to carina, red; bronchi, green. (a) Good segmentation, (b) acceptable segmentation, and (c) unacceptable segmentation.

The airway tree is segmented using a cylinder tracking and 3-D region growing-based method.^52^ A coronal view of the segmented airway (see Fig. 7) is used for the VEQR, where the trachea and the remainder of the airway tree are shown in different colors, thereby enabling the validation of the location of carina at the same time. Airway biomarkers are the airway diameter and wall thickness evaluated for each detectable segment. The grading criteria for the airway algorithm are: G: no visible errors; A: correct trachea and two main bronchi but unable to segment sufficient peripheral branches; and U: visible leakage.

##### Lungs

The left and right lungs are segmented using 3-D image filtering, intensity thresholding, and morphological operations.^66^ The left and right lungs are partitioned with a minimum distance path-cutting algorithm. The customized visualization is a 3-D rendering of the two lungs as shown in Fig. 5. Lung region biomarker evaluations include the detection of pulmonary nodules and the measurement of lung health indicators such as the emphysema index. The grading criteria for the lung algorithm are: G: no visible errors and U: major errors, e.g., another region mistaken as lung.

##### Individual vertebra

**Fig. 8:**
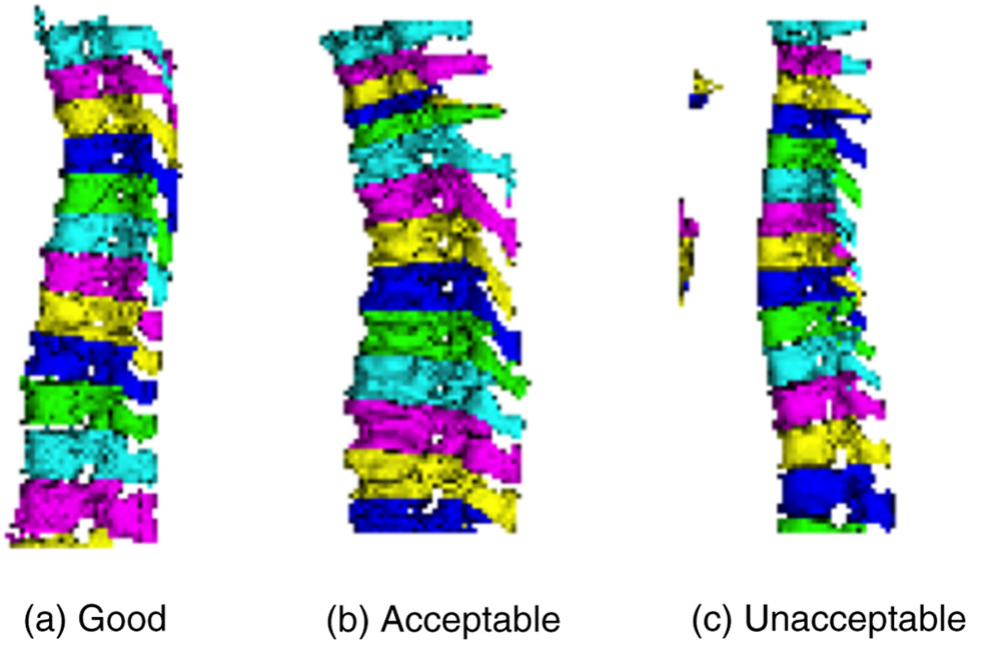
Customized visualization for segmentation of individual vertebra: individual vertebra colored differently. (a) Good segmentation, (b) acceptable segmentation, and (c) unacceptable segmentation.

The whole spine is first segmented by thresholding and connected component analysis; it is then further divided into individual vertebra by fitting separating planes in the 3-D space. VEQR of the individual vertebra segmentation is performed on the sagittal view of the 3-D segmentation (see Fig. 8), where different colors are used to indicate individual vertebra for the purpose of validating the vertebra labeling. Vertebra biomarker evaluations include the measurement of bone mineral density and the prediction of compression fracture. The grading criteria for the vertebra algorithm are: G: no visible errors: A: minor oversegmentation; and U: major errors including mislabeling or failure to separate vertebra.

##### Cardiac region

**Fig. 9:**
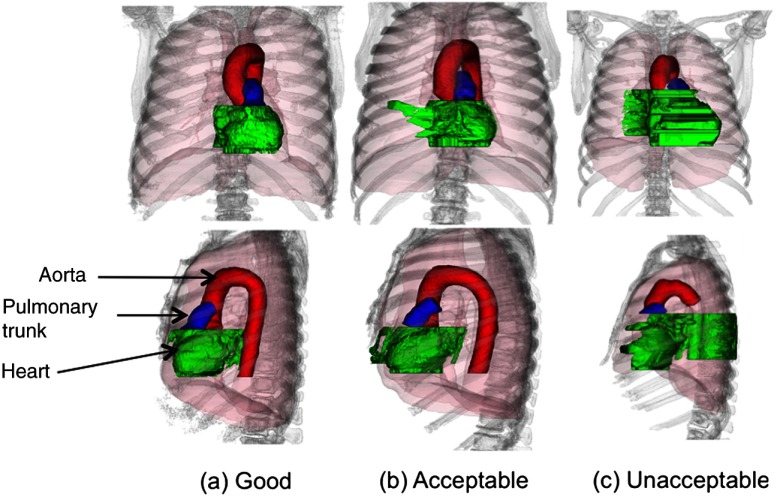
Customized visualization for cardiac region segmentation: aorta, red; heart, green; pulmonary trunk, blue. (a) Good segmentation, (b) acceptable segmentation, and (c) unacceptable segmentation.

Cardiac region segmentation includes the segmentation of aorta, heart region, and pulmonary trunk using anatomical constraint-based methods.^17^^–^^19^ VEQR of the cardiac region consists of the evaluation of coronal and sagittal views of the segmented aorta, heart region, and pulmonary trunk in different colors (see Fig. 9). Cardiac region primary biomarkers are CAC and aorta diameter profile. The grading criteria for the cardiac algorithm are: G: no visible errors in any of the three regions; A: visible errors but usable for measurement of biomarkers; and U: major errors and not usable for biomarker measurements.

##### Breast

**Fig. 10:**
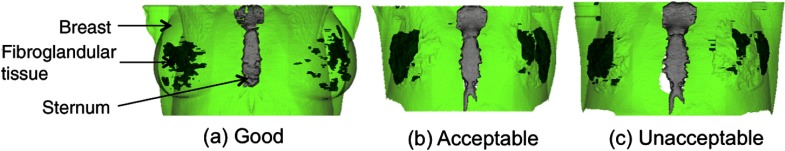
Customized visualization for breast segmentation: whole breast region (light green), fibroglandular tissue (solid green), and sternum (gray). (a) Good segmentation, (b) acceptable segmentation, and (c) unacceptable segmentation.

The whole breast region as well as the fibroglandular tissue and fat tissue contained in the breast region are segmented using an anatomy-oriented algorithm^21^ that separates the pectoral muscles from fibroglandular tissue through the propagation of muscle front along the vertical direction. A coronal view of the breast segmentation as shown in Fig. 10 is used for the VEQR, where the whole breast region and the fibroglandular tissue are shown in different colors. Breast biomarkers are breast density assessment and breast mass detection (ongoing project). The grading criteria for the breast algorithm are: G: no visible errors; A: minor segmentation issue, such as insignificant undersegmentation of fibroglandular tissue or inclusion of muscle into the breast region, which will not influence further biomarker analysis including density assessment and breast mass detection; and U: segmentation errors that may influence further biomarker analysis.

### 2.3 Large-Scale Image Annotation System Implementation and Evaluation

The large-scale image documentation method has been developed in conjunction with the chest health system application over the last 5 years with the dual goals of improving the robustness of the algorithms and creating a large documented dataset that may be used for the training and evaluation of other computer algorithms. We have used the current CHAS algorithms as the reference algorithm for the image documentation system and have conducted annual updates of first the CHAS algorithms and then the database image documentation. That is, each year algorithms are updated to address issues that caused some of the unacceptable segmentations, then the database is revised using the new CHAS algorithm set, and the reference algorithm used for new image data is set to the new CHAS algorithm set.

Several system parameters for use with the CHAS algorithms were empirically derived. For a visual inspection update to occur, the Dice coefficient difference between the new and reference segmentations must be between 0.75 and 0.95; that is, $T_{\mathrm{DC}\text{low}}=0.75$ and $T_{\mathrm{DC}\text{high}}=0.95$. A two-stage process was used for the introduction of new algorithms/biomarker tasks to the system. First the algorithms were carefully evaluated on a development dataset of 364 3-D images from public datasets, and the customized 3-D review was carefully reviewed. Further, a minimum performance level of 90% acceptable segmentations was required by the new algorithm before it was applied to the full dataset to prevent the generation of too many review events.

#### 2.3.1 Image cohorts

Six different cohorts of 3-D images from research studies were used for evaluation as outlined in Table 4. The image inclusion criteria are that the image parameters are consistent with LCS; that is, they are low-dose, thin-slice, noncontrast, supine position, and cover the whole lung region. The majority of the scans have been acquired with the I-ELCAP LCS protocol^67^ and have acquisition parameters: 120 kVp, <100  mA and slice thickness<=2  mm. Some of the LIDC cases used for algorithm development (179) exceed the low-dose requirement, some have 140 kVp, and the current has a range of 100 to 560 mA.

**Table 4:** System evaluation data cohorts 2016.

	Cases	Images	Images (include)	Images (exclude)	Slice thickness range (mm)
ELCAP	50	50	46	4 (8%)	1.25
LIDC	328	328	318	10 (3%)	<=1.25
NEW	1887	1892	1887	5 (0.3%)	2
WTC	1458	3795	3767	28 (0.7%)	<=1.5 (3763)
					2 (4)
FAMRI	710	1422	1422	0 (0%)	<=1.25
LC	2169	6764	6752	12 (0.2%)	<=1.25 (6733)
					1.5 to 2 (19)
Total	6602	14,251	14,192	59 (0.4%)	<=2

The six validation cohorts consist of (a) the VIA-ELCAP^68^^,^^69^ public dataset; (b) the LIDC^39^ public dataset and private research cohorts associated with the I-ELCAP study;^69^ (c) a nuclear energy workers dataset (NEW); (d) a never-smoker LCS dataset from a Flight Attendants Medical Research Institute (FAMRI) project; (e) a high-risk LCS cohort (LC); and (f) a research World Trade Center workers dataset (WTC). The public LIDC database contains over 1000 3-D images; however, only 328 3-D images met our inclusion criteria. The target slice thickness was 1.25 mm or less, with the only exception being the NEW cohort (2.0 mm). A single model SIEMENS scanner was used for the NEW cohort, a single model GE scanner was used for the ELCAP cohort, and all other cohorts involved multiple scanners. Several cohorts had multiple 3-D images per case; all 3-D images available were used for validation. A small fraction of the 3-D images were excluded from evaluation because of severe image artifacts caused by implants. The data cohorts that were available in 2016 are listed in Table 4. For 2017, we acquired additional data for the NEW, FAMRI, and LC datasets. The updated data cohort’s description is given in Table 5.

**Table 5:** System evaluation data cohorts 2017.

	Cases	Images	Images (include)	Images (exclude)	Slice thickness range (mm)
ELCAP	50	50	46	4 (8%)	1.25
LIDC	328	328	318	10 (3%)	<=1.25
NEW	1888	4678	4672	6 (0.1%)	2
WTC	1458	3795	3766	29 (0.8%)	<=1.5 (3762)
					2 (4)
FAMRI	932	2137	2137	0 (0%)	<=1.25
LC	2985	9820	9810	10 (0.2%)	<=1.25 (9804)
					1.5 to 2 (6)
Total	7641	20,808	20,749	59 (0.3%)	<=2

#### 2.3.2 Image documentation system evaluations

The overall performance of the system is indicated by number of documented 3-D images and the robustness (fraction of acceptable segmentations) by the CHAS algorithms. The main hypothesis is that the large-scale image documentation will facilitate the documentation of 1000’s of 3-D images with a success rate of acceptable segmentations of over 90% for each region type in a multiregion segmentation task. This implies a 90% success rate for the fully automated reference algorithm used to document the 3-D images. We present results for the last two (of five) cycles of operation of the system comprising of 14,192 and 20,749 3-D images, respectively.

Three additional aspects of the system were evaluated. First, the time for image evaluation of each 3-D image and the number of evaluation events triggered for an update are important cost factors in creating the image documentation. High-speed VE is important for constructing large-scale databases, the hypothesis is that for this documentation method the review time per scan will be only a few seconds rather than minutes and that the number of scans to be rereviewed for database updates will be a small fraction of the total. The time to document a dataset sample of 200 new sequential cases was monitored for all biomarker modules to identify the new data cost. For the updates time, two cohorts with 364 development cases, which had had several previous update reviews, and the 1422 FAMRI cases, for its first update review, were monitored for all biomarker-related modules. Second, the interreader variation was evaluated for two readers on the development dataset of 364 3-D images. High reader agreement is important for a high-quality image documentation. Using the 3-D visualization methods with well-defined categorical scores, the hypothesis is that there will be a very high interreader agreement for region evaluations. Finally, to evaluate on algorithms other than the CAHS algorithms, an external segmentation algorithm from the chest imaging platform (CIP)^49^ was evaluated on a subset of 7173 images. The documented dataset is designed for the efficient evaluation of computer algorithms. The hypothesis is that only a small fraction of images will require visual review when a new algorithm is evaluated with the documented dataset.

## 3 Results

Results on CHAS application evaluated by the SIMBA framework are presented as follows: (1) VE outcomes of each segmentation algorithm for years 2016 and 2017; (2) time estimates for the VE process; (3) interreader variation on the development dataset; and (4) comparison with an external lung segmentation algorithm.

### 3.1 Algorithm Evaluation Results

The VEQR results in the year 2016 to 2017 are given in Tables 6–9. Table 6 shows the VEQR results for the development dataset (364 CT 3-D images, agreement between two reviewers). Table 7 shows the combined VEQR results for the full 2016 dataset of 7440 cases (the additional 7076 CT 3-D images were reviewed by a single reviewer). In the 2016 year, the breast analysis algorithm was added to the CHAS system and a new cohort LC was added to the database. The results for the breast algorithm are also included in Tables 6 and 7. Table 8 shows the performance of the new cohort (LC, 6752 3-D images). After adding the additional 6557 3-D images in 2017, the complete result is reported in Table 9. For completeness, the results for the review of the two modules without specific biomarker objectives are also included as these were evaluated; they are ribs (24 labels) and skin surface (3 labels).

**Table 6:** VEQR results for VIA-ELCAP and LIDC 364 development 3-D images (2016/17, development dataset).

Grade	Airway	Lung	Ribs	Vertebra	Skin surface	Cardiac region	Breast
G	364 (100%)	364 (98%)	347 (95%)	300 (82%)	363 (99.7%)	333 (91%)	348 (96%)
A	0 (0%)	0 (0%)	10 (3%)	56 (15%)	0 (0%)	26 (7%)	5 (1%)
U	0 (0%)	0 (0%)	7 (2%)	8 (2%)	1 (0.3%)	5 (1%)	11 (3%)

**Table 7:** VEQR results for all datasets, 7440 3-D images (2016, baseline dataset).

Grade	Airway	Lung	Ribs	Vertebra	Skin surface	Cardiac region	Breast
G	7069 (95%)	7298 (98%)	6443 (87%)	6025 (81%)	7436 (99.9%)	6007 (81%)	6764 (91%)
A	88 (1%)	0 (0%)	489 (6%)	893 (12%)	0 (0%)	967 (13%)	361 (5%)
U	283 (4%)	142 (2%)	508 (7%)	522 (7%)	4 (0.1%)	466 (6%)	315 (4%)

**Table 8:** VEQR results for the new LC dataset of 6752 3-D images (2016 new dataset).

Grade	Airway	Lung	Ribs	Vertebra	Skin surface	Cardiac region	Breast
G	5554 (82%)	6675 (99%)	5389 (80%)	4616 (68%)	6736 (99.8%)	5202 (77%)	6141 (91%)
A	516 (8%)	0 (0%)	121 (2%)	674 (10%)	0 (0%)	444 (7%)	378 (6%)
U	682 (10%)	77 (1%)	1242 (18%)	1462 (22%)	16 (0.2%)	1106 (16%)	233 (3%)

**Table 9:** VEQR results for all datasets, 20,749 3-D images (2017, full dataset).

Grade	Airway	Lung	Ribs	Vertebra	Skin surface	Cardiac region	Breast
G	18,312 (88%)	20,443 (99%)	17,826 (86%)	15,423 (74%)	20,726 (99.9%)	17,673 (85%)	18,737 (90%)
A	908 (4%)	0 (0%)	884 (4%)	2828 (14%)	0 (0%)	1372 (7%)	894 (4%)
U	1529 (7%)	306 (1%)	2039 (10%)	2498 (12%)	23 (0.1%)	1704 (8%)	1118 (5%)

### 3.2 VE Timing Results

Table 8 shows the time cost for the VE procedure assessed on 200 sequentially selected 3-D images. With regard to the two-level viewing protocol, less than 2% of the module reviews required further inspection beyond the primary 3-D visualization. The second level 2-D visualization for these cases was ∼60  s. This time is included in the average times reported in Table 10.

**Table 10:** Average time cost per 3-D image for VE on each module.

Module	Airway	Lung	Ribs	Vertebra	Skin surface	Cardiac region	Breast
Time (s)	4.3	2.7	4.0	5.3	2.2	3.5	2.8

The comparative update review for the development dataset of 364 3-D images required evaluation of 2548 module outcomes. With the current acceptance thresholds, 26 module outcomes (1%) required side-by-side VE and six module outcomes (0.2%) resulted in database updates. For the FAMRI dataset of 1422 3-D images (9954 module outcomes), 187 (19%) of the module outcomes from 185 3-D images required side-by-side VE and 99 module outcomes (1%) resulted in database updates. The side-by-side comparison between reference segmentation and new segmentation for all 187 FAMRI dataset reviews required 21 min; that is an average of 6.7 s per module outcome review.

### 3.3 Interreader Variation

Two readers visually reviewed the development dataset of 364 3-D images independently. The same grading criteria were used for each segmentation, and the differences between the readers are summarized in Table 11. A difference occurs if one reader has assigned a grade of G or A while the other reader has assigned a grade of U to the same segmentation.

**Table 11:** Difference between two readers (364 development 3-D images).

Module	Airway	Lung	Ribs	Vertebra	Skin surface	Cardiac region	Breast
Differences (images)	0	0	0	1	1	2	2

### 3.4 Evaluation Results for the External Lung Segmentation Algorithm

An external lung segmentation algorithm as part of the CIP,^49^ which is an open-source library for quantitative image analysis on lung and airway,^70^ was evaluated through the presented SIMBA system with a subset of 7173 CT scans from the full database. Visual inspection was triggered on 74 scans, including 26 scans in which the DC between the external algorithm and the reference algorithm were in the range $\left[T_{\mathrm{DC}\text{low}},T_{\mathrm{DC}\text{high}}\right]$, with $T_{\mathrm{DC}\text{low}}=0.75$ and $T_{\mathrm{DC}\text{high}}=0.95$, and 48 scans for which the reference algorithm had failed to produce acceptable segmentation. No visual inspection was needed for 7099 scans, including 12 scans in which the DC were below $T_{\mathrm{DC}\text{low}}$ and 7087 scans in which the DC were above $T_{\mathrm{DC}\text{high}}$. The unacceptable reference segmentations for 37 scans (0.52% of the total evaluated) were replaced by the new algorithm acceptable outcomes.

## 4 Discussion

The large-scale image documentation method has been used to document over 20,000 3-D images with over 100 labeled regions. We note that far fewer regions are currently used for biomarker evaluation: lung (2 regions), airway (2 regions), vertebra (15 regions), cardiac (3 regions), and breast (6 regions). Depending on the region, from 88% to 99% of these 3-D images have acceptable region segmentations as shown in Table 9.

### 4.1 CHAS Segmentation Algorithm Performance

The results on the development dataset in Table 6 show very high success rates (98% to 100%) acceptable for the more mature modules that have received several cycles of revision compared to the more recent modules. Even the new breast module has 97% acceptable. These modules’ development have all benefited from some extreme cases extracted from the larger full dataset in addition to the public development dataset. In Table 7, the results for the full 2016 dataset of 7440 3-D images show that the acceptable rate is from 93% to 99%, which is within our initial target of at least 90%.

Table 7 shows the initial results on the newly added 6752 3-D images. The ribs and vertebrae modules have an acceptance rate of 82% and 78%, respectively, which is below our 90% target for new algorithms and resulted in low success rates for other modules. Review of a subset of these cases showed that this cohort had scanner parameters that resulted in different noise statistics than our previous cohorts and that the effect was to cause our bone segmentation algorithms to give poor results. After revision, our algorithms now detect this noise characteristic in the image quality analysis module (Fig. 6), and filter the image data prior to bone segmentation. The results for the upgraded system and algorithms for 2017 are shown in Table 9, in which an additional 6557 3-D images were added to the database. The results for 2017 indicate that the performance of the updated CHAS has improved even with the additional image and with only one module (vertebra, 88%) not achieving the 90% target.

### 4.2 Visual Image Evaluation Effort

Results given in Table 10 show that the per 3-D image evaluation time for the initial VE is about 25 s. The time for just nodule review by a radiologist for these scans without CAD has been shown to be in the order of 10 min.^71^^,^^72^ Therefore, the time for initial segmentation review with the SIMBA framework is a tiny fraction (<5%) of the time required for a traditional clinical review of the 3-D image. In addition, update reviews are required when the outcomes from new algorithms are considered. Currently, we are observing about 1% to 2% module reviews that require a review time of about 7 s. This total cost of updating is linearly related to the size of the database; however, as the algorithms mature, it is anticipated that the fraction of triggered events will significantly diminish; note the lower frequency of trigger events for the development dataset compared to more recent FAMRI dataset.

The more mature algorithm modules in CHAS have now received several cycles of updates. For example, when our standard lung module was applied to the LIDC 318 cases, the initial performance was about 70%. With several cycles of algorithm refinements, the performance on that dataset is now 97% to 100%, and attention is directed to failures in the other cohorts.

### 4.3 Interreader Variation

The limited study on interreader variation indicated that there were a total of only six 3-D image evaluations out of 2548 (seven algorithms for 364 3-D images), i.e., about 0.24%. Further, the six 3-D images in question were considered to be close to the border of acceptable. This is in line with our design goal of rating the segmentation for the relevant task biomarker evaluation rather than traditional boundary precision. A down side to this approach is that if a new quantitative biomarker is added that requires new segmentation characteristics, then the entire database may need to be re-evaluated.

### 4.4 Algorithm Evaluation

The evaluation of the CIP lung segmentation algorithm^49^ demonstrates that the documented database may be used for evaluating different algorithms. Visual review was only required on 74 cases; that is 1.03% of the testing dataset. Although the system does not support the cloud-based approach used by Visceral^36^ for algorithm evaluation, it is simple to include an external algorithm module in the SIMBA system; further, it is possible to export images for testing and import the resulting segmentations for evaluation.

### 4.5 Limitations of Study

The large-scale image documentation method has been demonstrated with a single application and a single validation method (visual inspection with grading into three categories and no manual marking). There are many alternative strategies that could be used with this framework. For example, a cohort of phantom images with *a priori* known truth can easily be added to the system. A second possibility is to increase inclusiveness by using other methods, such as manual marking to provide good segmentations for the cases graded unacceptable. The tradeoff for this approach is a much higher cost in documentation effort combined with the introduction of human variation into the image documentation. Adding manual documentation for difficult cases may be very useful, especially in scenarios where the database is to be used directly for algorithm training. In addition to the multiregion application discussed here, the framework has also been successfully used on other research projects involving just one or two regions.

Currently, there are some image quality checks within the algorithm modules that indicate algorithm failure and prevent dependent algorithms from executing. In future work, we plan to enhance outcome quality checks such that, for many cases, an automatic unacceptable evaluation grade will be given when a problem is detected. This mechanism will further reduce the number of images that require VE for both new image data and for database updates.

In this work, we have not established that the chosen VE is equivalent or superior to other evaluation methods. A major challenge in evaluating the quality of computer segmentations for medical imaging is that, in general, the true value is unknown and that comparison must be made by either QE or VE methods. Traditionally, radiologists view and evaluate 3-D images by a sequence of 2-D image slices extracted along the axial dimension. More recently with the advent of electronic image viewing systems, other views including sagittal, coronal, MIP projections, and 3-D visualizations are becoming more common. In this work, we take advantage of a well-defined anatomical region possibly being more rapidly evaluated using primarily several 3-D visualizations rather than reviewing every axial 2-D image slice. We have not established any specific precision advantage for any of these approaches; however, we conjecture that different methods may provide acceptable outcomes.

### 4.6 Demonstration SIMBA Web System

The image documentation system images and segmentation visualizations have been made available on a demonstration web system.^5^ This allows for access to the ELCAP-VIA public image database of 50 LDCT chest 3-D images and the resulting CHAS image segmentations. In addition, the web system supports interactive viewing of CT 3-D images and the associated customized visualizations for both the initial image review and for the side-by-side update image review for CHAS database documentation.

## 5 Conclusion

A large-scale image documentation method facilitates the efficient segmentation image documentation by incorporating the VEQR method and customized visualizations. The efficacy of this method has been demonstrated with the documentation of seven main regions and over 100 subregions in over 20,000 3-D images for the CHAS image analysis system. For the main regions, the success rate varied from 88% to 99.8% (median 93%).

The key concept for realizing a practical documentation system is minimizing the VE time; this has been accomplished by a focus on image segmentation and two main methods: (a) customized biomarker-inspired image visualization and (b) the VEQR protocol. Further, this approach avoids both the high cost and inherent error through human variation incurred using manual marking methods. This method has the potential to be used for the documentation of extremely large image databases.

This image documentation method addresses a pressing need for the documentation of very large image databases needed for (a) computer algorithm development, (b) the training of emerging machine learning methods, and (c) supporting clinical research (both to understand natural disease history and to support clinical studies) by facilitating the collection of biomarker statistics on large population cohorts. The timing results for segmentation VE of a few seconds for each organ indicate that building validated image databases can be achieved at a very small incremental cost of effort compared to that of a traditional clinical image read by a radiologist.

Performance results for the CHAS application indicate that a high proportion of segmentation success (93% acceptable segmentations in over 20,000 cases) is achievable on LDCT 3-D images; further, the review of segmentation quality only requires a few seconds of effort. This suggests that automated evaluation of several important lung health measures with visualizations for rapid review may be useful for clinical practice in a LCS setting. Future plans include applying this method to an LCS registry that includes image data.
